# The HERC1 ubiquitin ligase regulates presynaptic membrane dynamics of central synapses

**DOI:** 10.1038/s41598-020-68970-8

**Published:** 2020-07-21

**Authors:** Mª Angeles Montes-Fernández, Eva Mª Pérez-Villegas, Francesc R. Garcia-Gonzalo, Leonardo Pedrazza, Jose Luis Rosa, Guillermo Alvarez de Toledo, José A. Armengol

**Affiliations:** 1 Department of Medical Physiology and Biophysics, School of Medicine, University of Seville, Seville, Spain; 2 Department of Physiology, Anatomy and Cell Biology, University Pablo de Olavide, Seville, Spain; 3 Department of Physiological Sciences, IDIBELL, University of Barcelona, Barcelona, Spain

**Keywords:** Neurotransmitters, Synaptic vesicle endocytosis

## Abstract

HERC1 is a ubiquitin ligase protein, which, when mutated, induces several malformations and intellectual disability in humans. The animal model of HERC1 mutation is the mouse *tambaleante* characterized by: (1) overproduction of the protein; (2) cerebellar Purkinje cells death by autophagy; (3) dysregulation of autophagy in spinal cord motor neurons, and CA3 and neocortical pyramidal neurons; (4) impairment of associative learning, linked to altered spinogenesis and absence of LTP in the lateral amygdala; and, (5) motor impairment due to delayed action potential transmission, decrease synaptic transmission efficiency and altered myelination in the peripheral nervous system. To investigate the putative role of HERC1 in the presynaptic dynamics we have performed a series of experiments in cultured *tambaleante* hippocampal neurons by using transmission electron microscopy, FM1-43 destaining and immunocytochemistry. Our results show: (1) a decrease in the number of synaptic vesicles; (2) reduced active zones; (3) less clathrin immunoreactivity and less presynaptic endings over the hippocampal main dendritic trees; which contrast with (4) a greater number of endosomes and autophagosomes in the presynaptic endings of the *tambaleante* neurons relative to control ones. Altogether these results show an important role of HERC1 in the regulation of presynaptic membrane dynamics.

## Introduction

HERC1 is a giant phylogenetically conserved ubiquitin ligase of the HECT family^1^ that participates in the ubiquitin–proteasome system (UPS)^2–3^. Like other UPS alterations, mutations in HECT E3 ligases have been associated with the pathogenesis of neuromuscular disorders, Parkinson’s disease and diseases of the autism spectrum^1–4^. Furthermore, mutations in the RCC1 domain of human HERC1 have been related with X-linked *retinitis pigmentosa* and juvenile amyotrophic lateral sclerosis 2^5^. In humans, missense mutations of *Herc1* display polymorphic syndromes with or without cerebellar affectation^6–8^, in which the intellectual disability appears as the common neurological disorder^8^.

The *tambaleante* (*tbl*) mutant mouse was earliest reported as a model of adult cerebellar ataxia caused by the almost complete autophagy cell death of cerebellar Purkinje cells^9–11^. In addition to adult cerebellar Purkinje cell degeneration^5,9–12^, other alterations in the central and the peripheral nervous system have been recently described in *tbl* mouse such as: (1) increase of autophagy signs in spinal cord motor neurons and neocortical and CA3 hippocampal pyramidal neurons^13^; (2) impairment of the associative learning associated to absence of long term potentiation (LTP), altered dendritic spinogenesis, and a drastic decrease of glutamatergic innervation of the lateral amygdala^14^; (3) anomalous myelination in the sciatic nerve together with alterations of non-myelinating terminal Schwann cells at the neuromuscular junction (NMJ)^15^; and, (4) altered motor performance owing to a reduction of the motor end-plate area, and impaired evoked neurotransmitter release at the NMJ^16^. Molecular studies identified that HERC1 mutation carried by the *tbl* mice, and responsible for autophagy cell death, is a Gly483Glu spontaneous substitution located in the N-terminal RCC1 domain (RLD1)^5^. This domain acts as guanine nucleotide-release factor for ARF proteins and influences intracellular vesicle trafficking interacting with ARF/Rab GTPases^1–2^. Furthermore, C-terminal RCC1 domain (RLD2) of HERC1 forms a ternary complex with clathrin (CLT) and the heat shock protein 70, and might influence intracellular vesicular trafficking^17^.

These data together strongly suggest that HERC1 mutation of *tbl* mouse might alter the normal dynamic of excitatory presynaptic terminals. Furthermore, CLT mediated endocytosis (CME) is a key step for synaptic vesicle recycling^18^; thus, alterations of HERC1-CLT interaction^1,17^ might alter the normal CLT cycle interfering with the normal synaptic function. Therefore, to elucidate the putative role of HERC1 in the synaptic vesicle populations of central excitatory synapses and in their presynaptic dynamics, we have analyzed *tbl* hippocampal neuronal cultures in vitro by using transmission electron microscopy, immunocytochemical, GFP pull-down and FM1-43 destaining methods.

## Results

**Figure 1:**
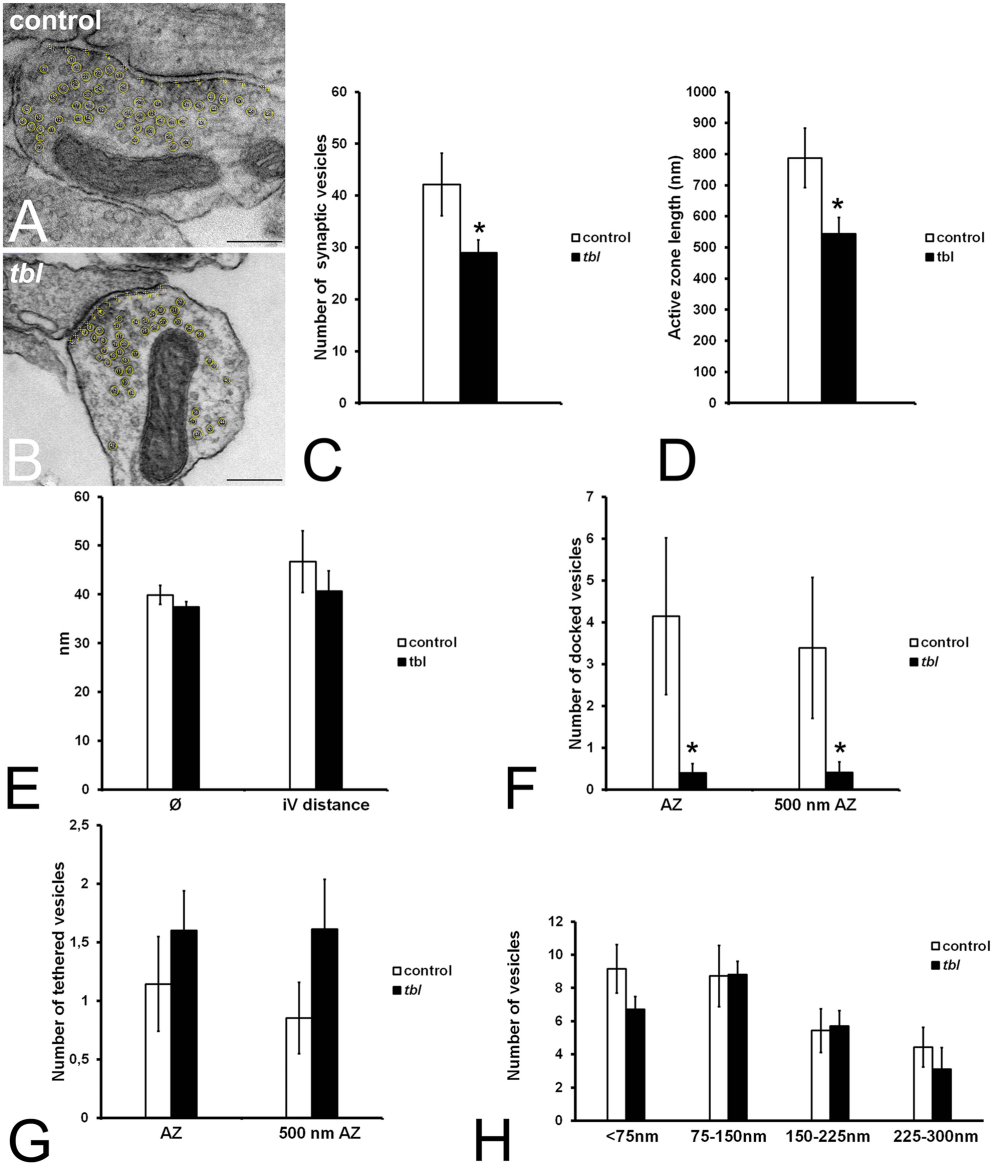
Electron microscopy microphotographs illustrating the ImageJ counts of the number of synaptic vesicles in control (**A**) and *tbl* (**B**) hippocampal cultures. The active zone is consistently shorter in *tbl* than in control presynaptic boutons (**D**; *p = 0.0246). Quantitative analyses demonstrate that *tbl* synapses possess less synaptic vesicles than control ones (**C**; *p = 0.0391), and fewer docked synaptic vesicles in both absolute (**F** AZ, *p = 0.0470) and normalized values (**F**; 500 nm AZ, *p = 0.0265). However, no significant statistic differences are found in the synaptic vesicles diameter (**E**; ∅, *p* = 0.1452), the intervesicular distance (**E**, iV distance, *p* = 0.2184), in the number of tethered vesicles (**G**; AZ, *p* = 0.2011; 500 nm AZ, *p* = 0.0834), and in the number of vesicles respecting their distance from the active zone (**H**; < 75 nm, *p* = 0.0853; 75–150 nm, *p* = 0.4834; 150–225 nm, *p* = 0.4342; 225–300 nm, *p* = 0.2318). Bars = 200 nm.

In present experiments those synapses of control (Fig. 1A) and *tbl* (Fig. 1B) hippocampal cultures showing a clear synaptic cleft, and evident thickening of pre- and postsynaptic zones were only considered for vesicle counts and active zone length measurement. The number of round and clear synaptic vesicles counted and the active zone length were significantly fewer (Fig. 1C) and shorter (Fig. 1D) in *tbl* synapses relative to control ones. However, significant differences were neither found in the mean diameter of the synaptic vesicles (Fig. 1E,∅) nor in the intervesicular distance (Fig. 1E) between control and *tbl* synapses. Furthermore, there was no statistically significant differences in the number of tethered synaptic vesicles (Fig. 1G); and, although the numbers of vesicles located in the nearest (< 75 nm) and farthest (225–300 nm) compartments of *tbl* synapses were fewer than in control ones, their values were not significant (*p* = 0.0853 and *p* = 0.2318 respectively) (Fig. 1H). However, a clear significant statistical difference in the number of docked vesicles was found, whose number was ever higher in control synapses relative to in *tbl* ones –as much in absolute values (Fig. 1F, AZ) as in normalized values relative the mean value of *tbl* active zone values (Fig. 1F, 500 nm AZ).

**Figure 2:**
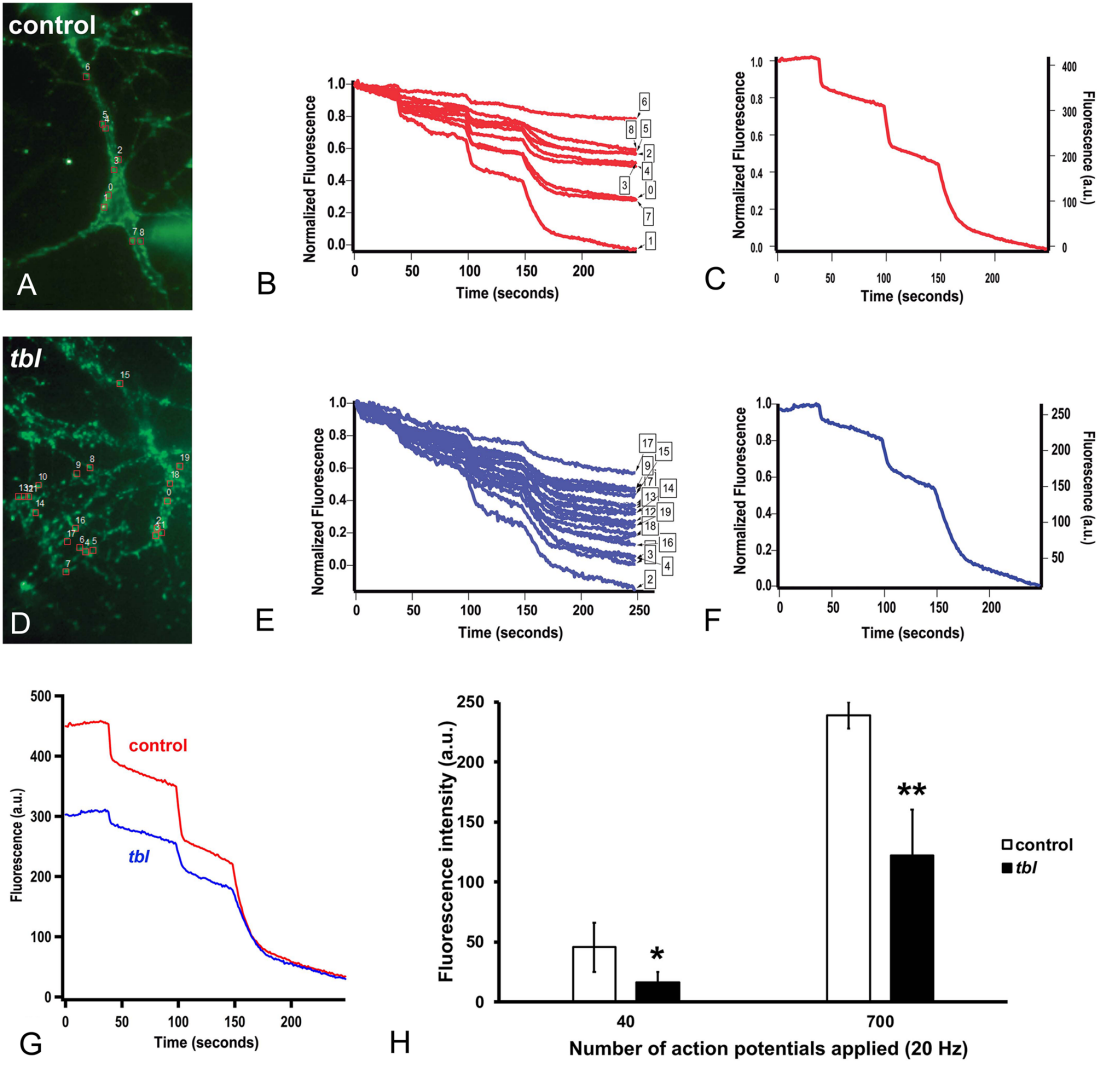
FM1-43 destaining of control and *tbl* hippocampal neurons in culture with successive applications of 40 APs, to deplete the RRP, and 100 plus 600 APs to deplete the reserve pool of synaptic vesicles. (**A**), control neuron stained with FM1-43. The ROIs are shown in red. (**B**), time course of FM1-43 destaining for multiple boutons. (**C**), Average FM1-43 destaining (left axis normalized fluorescence, right axis total fluorescence. (**D**), *tbl* hippocampal neuron stained with FM1-43. ROIs marked in red as in (**A**). (**E**,**F**), time course of FM1-43 destaining for *tbl* hippocampal neurons for individual boutons (**E**) and the average response (**F**). (**G**), superposition of control and *tbl* FM1-43 response for comparison. Note that control neurons were better loaded than *tbl* mutant neurons. (**H**), both, the RRP and the size of the reserve pool of synaptic vesicles were smaller in the *tbl* mutant, suggesting a lower size of dock and recycle vesicles. The asterisks indicate significant differences in the Student’s *t* test: ***p = 0.04).

To quantify synaptic vesicles pools we use the fluorescence lipophilic dye FM1-43, that label synaptic boutons in an activity-dependent manner that involves dye uptake by synaptic vesicles that are recycling^19^. Hippocampal cultures of control and *tbl* mutants were loaded with FM1-43 dye after the application of 600 pulses (Fig. 2A and D). The amount of FM1-43 loaded depends on the recycling activity of the neuron. Control cultured neurons revealed a greater amount of fluorescence as compared to *tbl* ones (450 ± 40; n = 6, vs 250 ± 35; n = 5) indicating larger dye loading. Unloading of FM1-43 of individual boutons for control and *tbl* revealed a large variability in both animals, reflecting a diversity of individual boutons in the amount of dye loaded (Fig. 2B and E). A set of 40 and 700 pulses were sufficient to unload dye completely in both control and *tbl* cultured neurons (Fig. 2C and F). A comparison of the destaining of FM1-43 for the control and *tbl* is shown in Fig. 2G. Quantification of the fluorescence change upon different levels of stimulation is shown in Fig. 2H. The application of 40 pulses produced a decrease of 45.7 ± 20.4 fluorescence units in the control (n = 4) *versus* 16.1 ± 8.9 in the *tbl* mutant (n = 4). This fluorescence change corresponds to approximately 9 vesicles in the RRP for the control and 3 for the *tbl* mutant, since previous experiments in the same set up revealed 5 fluorescence units per synaptic vesicle^20^. The application of 700 consecutive pulses, sufficient to deplete the reserve pool, showed also a significant difference between the control and the *tbl* mutant hippocampal cultured neurons (238.9 ± 108 *vs*. 122 ± 51.3; n = 100 and n = 75 boutons, *p* = 0.007).

**Figure 3:**
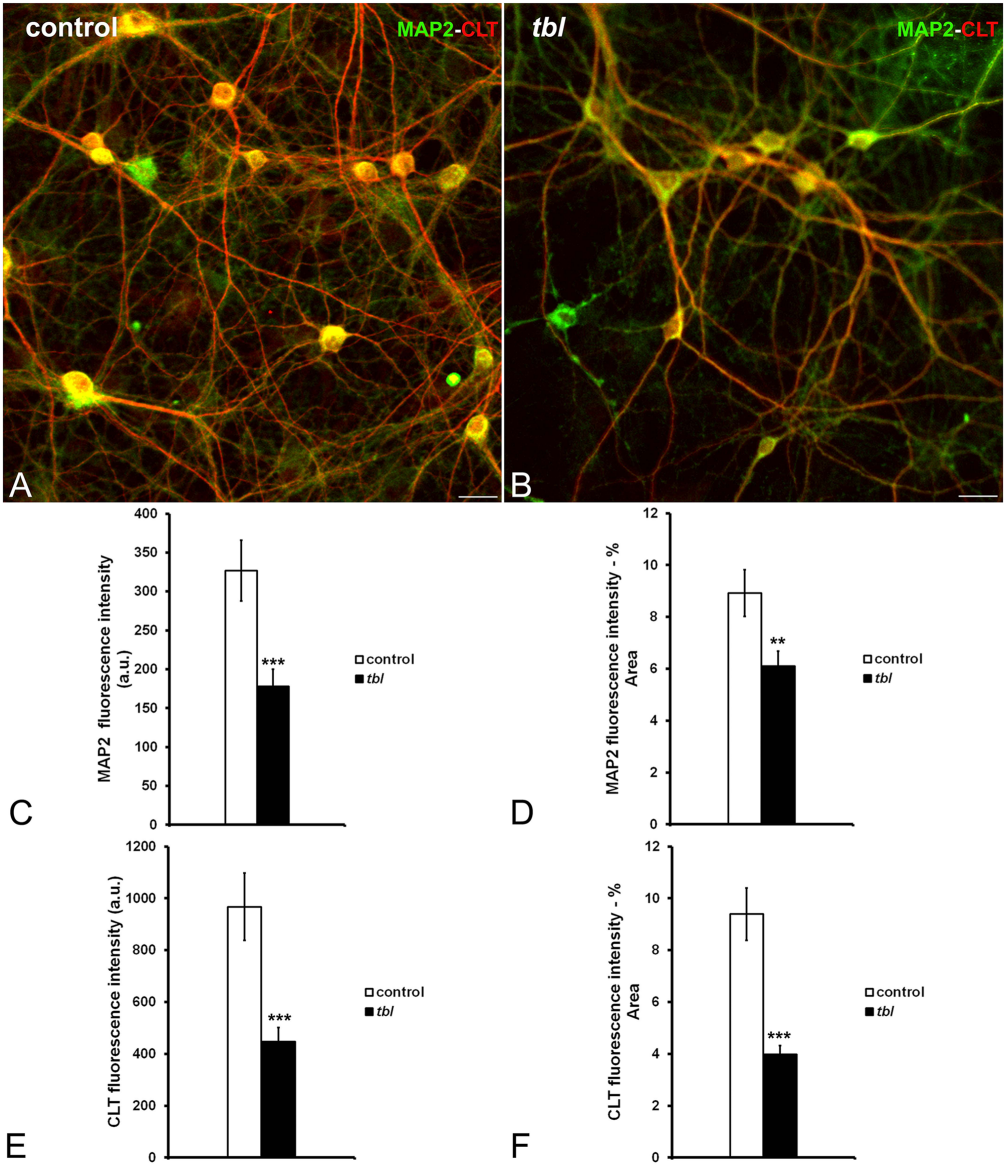
Laser confocal microphotographs of hippocampal cultures from control (**A**), and *tbl* (**B**) mice illustrative of MPA2 (green) and CLT (red) immunocytochemistry. (**C**–**F**), graphical representation of the fluorescence intensities of MAP2 and CLT in absolute values per image (**C**,**E**); and relative to the % of measured area (**D**,**F**). In both absolute (**C**,**E**) and relative (**D**,**F**), values, MAP2 and CLT expressions are higher in control cultures than in *tbl* ones. The asterisks indicate significant differences in the Student’s *t* test: ***p = 0.0008 (**C**); **p = 0.005 (**D**); ***p = 0.0005 (**E**); and ***p = 4.4^E−06^ (**F**). Bars = 50 µm. a.u., arbitrary units.

**Figure 4:**
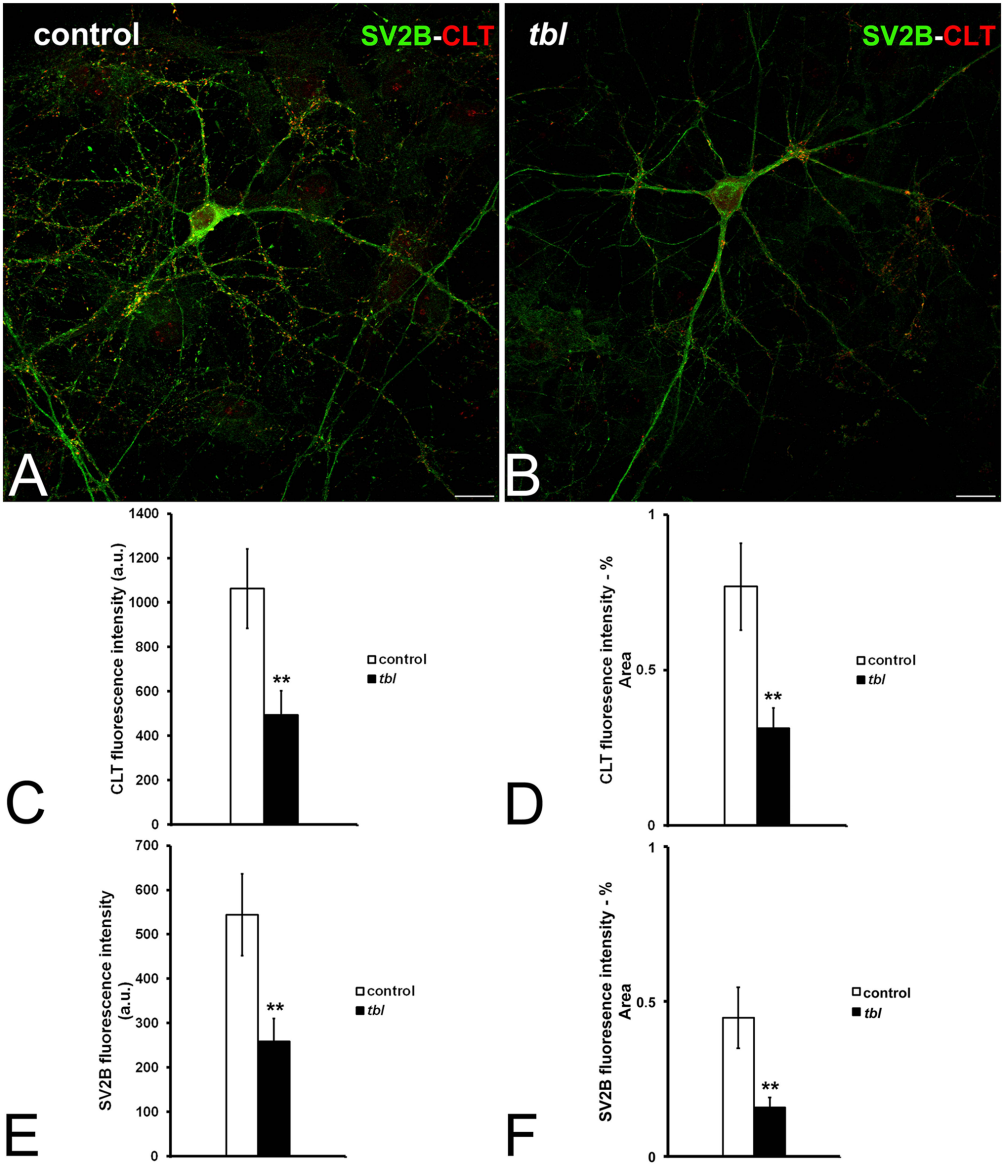
Laser confocal microphotographs illustrative of SV2B (green) and CLT (red) immunoreactivities in control (**A**) and *tbl* (**B**) hippocampal cultures. (**C**–**F**), graphs showing the fluorescence intensities of CLT (**C**, **D**) and of synaptic vesicles identified by SV2A immunoreactivity (**E**,**F**). In both absolute and % relative values, fluorescence intensity was higher in control than in *tbl* cultures (**C**, **p = 0.004; **D**, **p = 0.002; **E**, **p = 0.004; **F**, **p = 0.003). Bars = 30 µm. a.u., arbitrary units.

**Figure 5:**
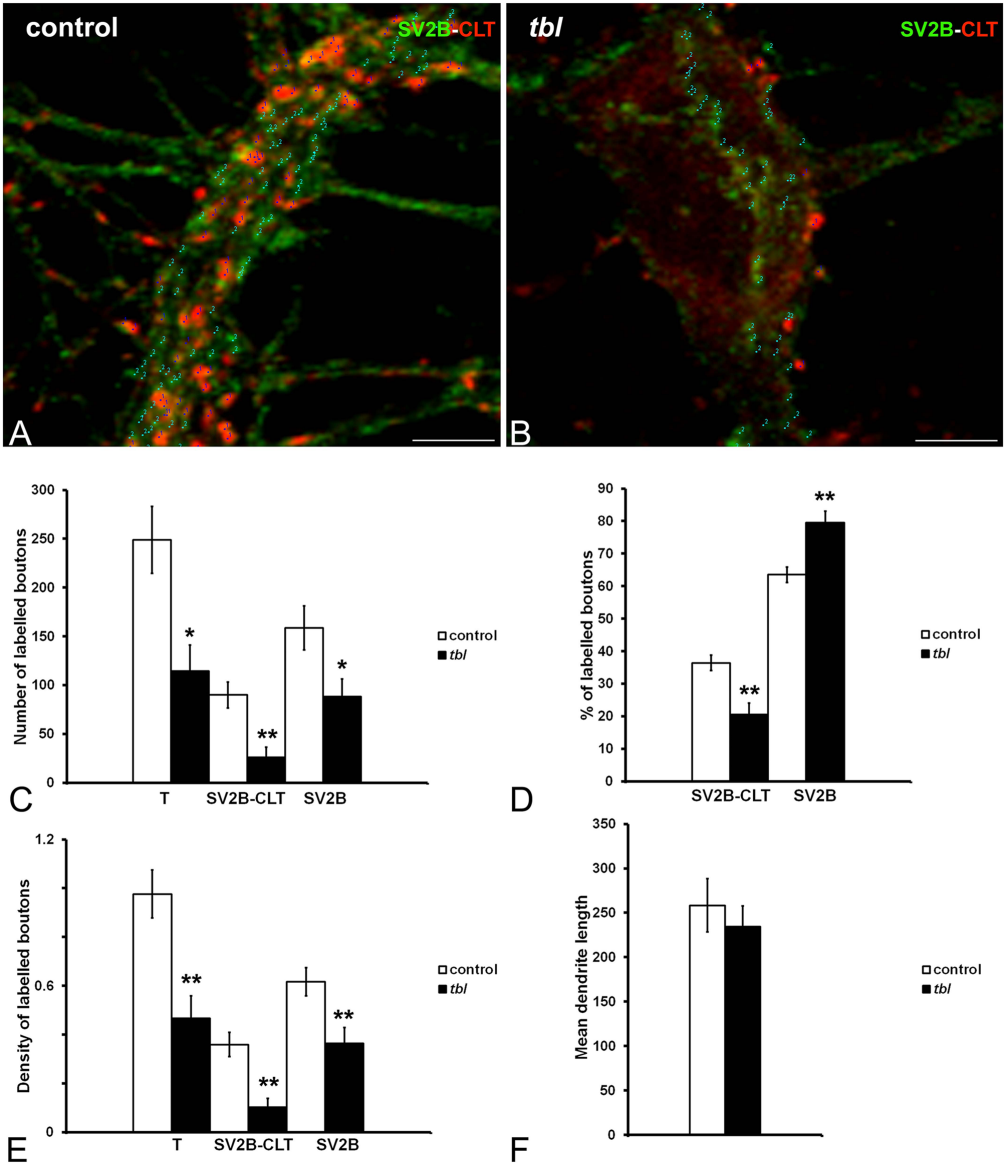
ImageJ composites illustrative of the count of presynaptic boutons expressing SV2B (green), CLT (red), or both immunoreactivities in control (**A**) and *tbl* (**B**) hippocampal cultures. (**C**–**E**), graphs showing the number of single SV2B and double (SV2B-CLT) labelled boutons (**C**), the percentage of labelled boutons (**D**), and their density of counted boutons *per* dendrite µm (**E**). The number of presynaptic boutons is higher in control relative to *tbl* cultures (**C**, T: *p = 0.014; SV2B-CLT: **p = 0.005; SV2: *p = 0.04). Correlative to these counts, the percentage of double labelled boutons is lower in *tbl* cultures relative to control ones (**D**, SV2B-CLT: **p = 0.007). The density of the number of all categories of presynaptic boutons counted is ever lower in *tbl* cultures than in control ones (**E**, T: **p = 0.005; SV2B-CLT: **p = 0.003; SV2: **p = 0.009), while no significant differences were found on the mean of the dendrite’s length measured (**F**; *p* = 0.544). Bars = 5 µm. T, total number of counted boutons.

**Figure 6:**
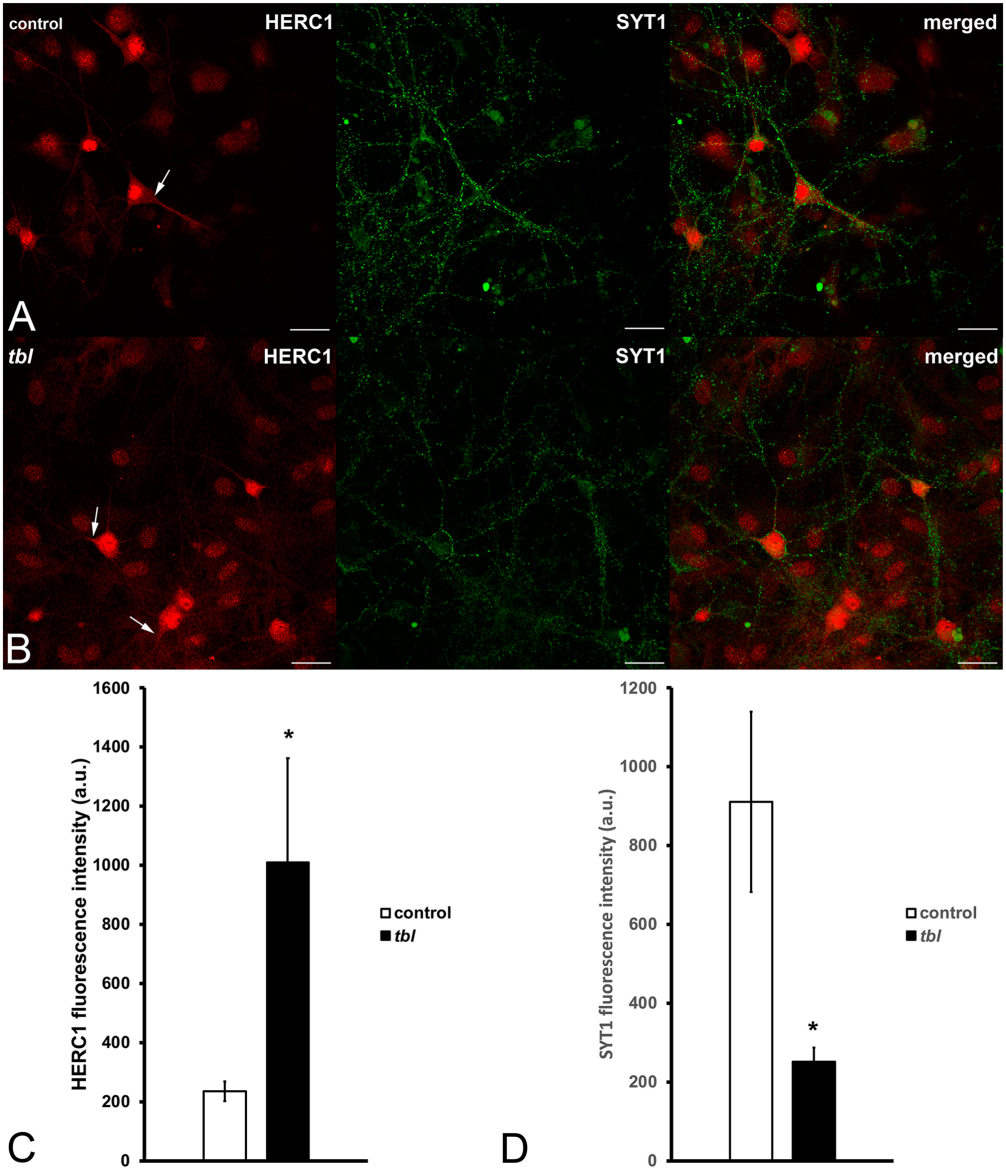
Laser confocal microphotographs illustrative of HERC1 (red) and SYT (green) immunoreactivities in control (**A**) and *tbl* (**B**) hippocampal cultures. HERC1 is expressed in both the neuronal nucleus (**A**–**B**, n) and cytoplasm (**A**–**B**, arrows). (**C**–**D**) graphs showing the fluorescence intensities of HERC1 (**C**) and of presynaptic terminal endings identified by SYT (**D**). Herc1 expression is greater in *tbl* cultured neurons relative to control ones (C, *p = 0.04723). In contrast, SYT immunoreactivity is considerably higher in control cultured neurons compared to *tbl* ones (**D**, *p = 0.04651). Bars = 5 µm.

Cultured *tbl* hip^21^pocampal neurons seem not show evident qualitative differences in size relative to control ones. The use of microtubule associated protein 2 (MAP2) as neuronal dendritic marker allowed us to analyze the neuronal expression of CLT (Fig. 3A-B). Control hippocampal neurons expressed strongly both markers relative to *tbl* ones; and the quantitative analyses demonstrated the existence of significant differences in the absolute (Fig. 3C and E) and normalized values relative to the analyzed area (Fig. 3D and F) of MAP2 and CLT expression. As CLT plays a key role in the synaptic vesicle endocytic recycling^22^, we have analyzed the immunocytochemical expression of CLT together to the presynaptic SV2B protein as general marker of synaptic vesicles (Fig. 4A-B). The expression of both markers was significantly higher in control cultured neurons than in *tbl* ones (Fig. 4C-F). Moreover, the counts of presynaptic boutons expressing SV2B alone or co-expressing with CLT (Fig. 5A-B) clearly indicated that the number of presynaptic boutons lying over the main dendrites of control hippocampal neurons analyzed here was higher than those ending on the main dendrites of *tbl* hippocampal neurons (Fig. 5C, T); this significant difference in the number of synaptic boutons is consistent from both SV2B single (Fig. 5C, SV2B) and double labelled boutons (Fig. 5C). When these numbers were normalized regarding their density relative to each µm of dendritic shaft measured, difference clearly ratifies that the density of synaptic boutons ending on the main dendrite of *tbl* hippocampal neurons was the half in relation to the control (Fig. 5E). The reduction in the density of presynaptic boutons is also correlated with a significant decrease in the immunolabelling of synaptotagmin 1 (SYT1), a presynaptic ubiquitous protein^22^, in *tbl* cultured neurons in relation to control cultured neurons (Fig. 6D).

**Figure 7:**
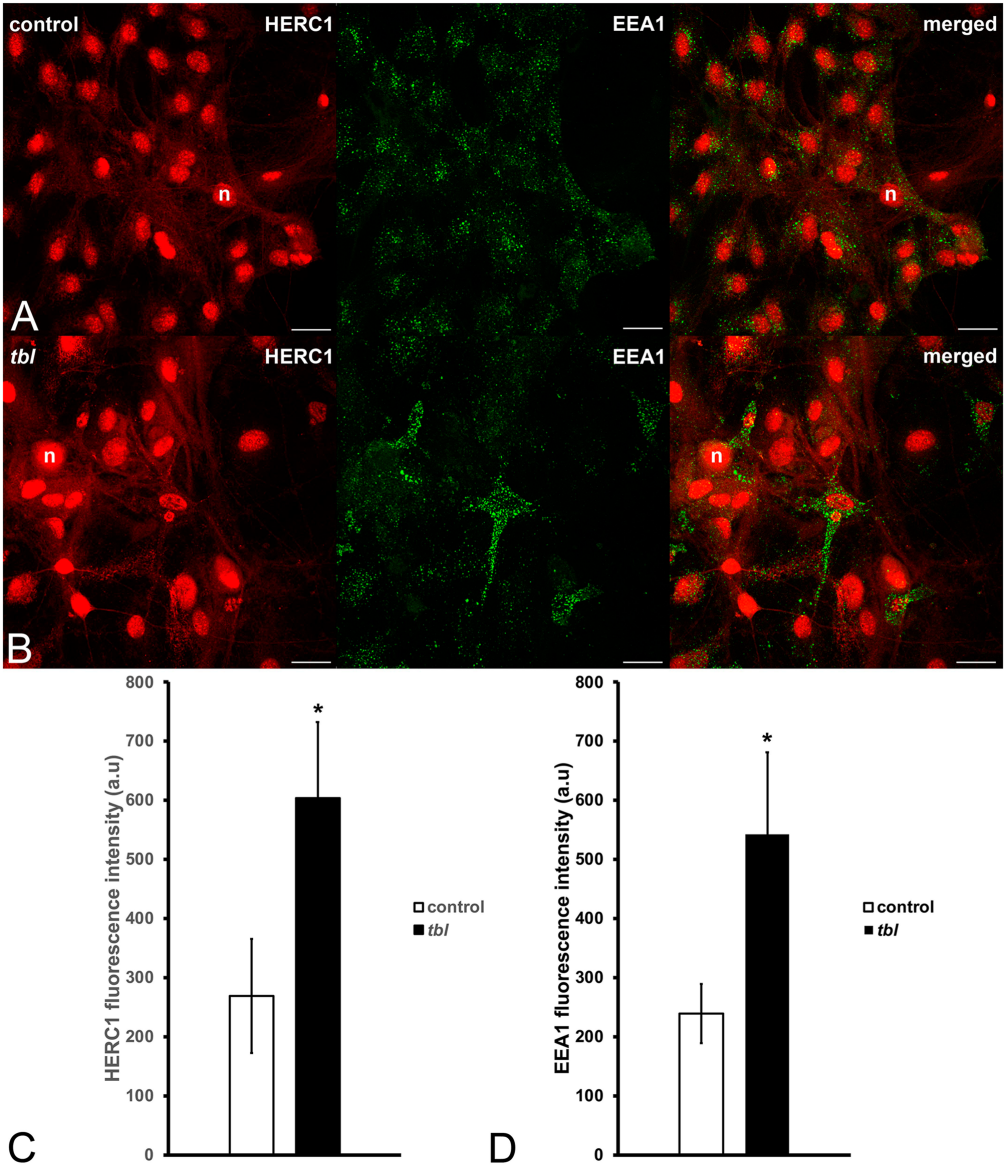
ImageJ composites illustrative of the expression of HERC1 (red) and EEA1 (green), in control (**A**) and *tbl* (**B**) hippocampal cultures. (**C**–**E**), graphs show the intensity of the expression of HERC1 (**C**) and EEA1 (**D**). HERC1immunoreactivity is higher in *tbl* relative to control cultures (**C**, *p = 0.0404). Likewise, EEA1 expression is lower in control cultures relative to *tbl* ones (**D**, *p = 0.04276). Bars = 5 µm. n, neuronal cell nucleus.

In our hands the commercial antibody used here against the HERC1 in hippocampal cultures (Figs. 6 and 7) shows that the expression of this protein is distributed through the cell nucleus and the cytoplasm (Figs. 6A-B and 7A-B). In all analyzed cultures, HERC1 expression was significantly more intense in *tbl* hippocampal neurons than in controls (Figs. 6C and 7C).

**Figure 8:**
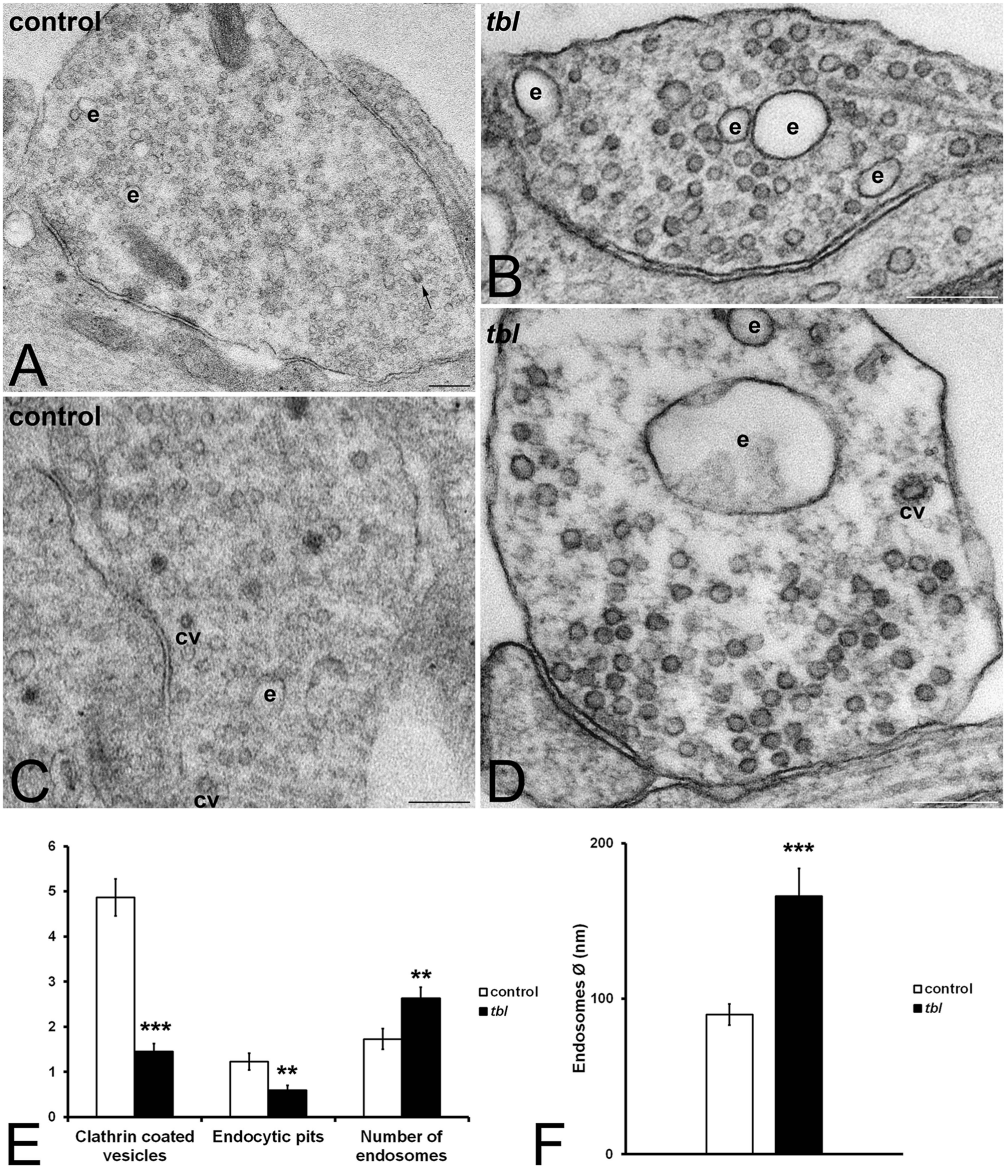
Electron microphotographs illustrating the main differences between control (**A**,**C**) and *tbl* (**B**,**D**) coated vesicles (cv) and endosomes (e). Coated vesicles and endocytic pits are most numerous in control than in *tbl* synaptic boutons (**E**, ***p = 2.559^E−08^; **p = 0.00936, respectively). Otherwise, endosomes were most numerous (**E**, **p = 0.00543) and greater (**F**, ***p = 0.00016) in *tbl* than in control presynaptic endings. The arrow in A indicates a coated vesicle integrating into an endosome. Bars = 200 nm.

Apart the differences in the number of rounded vesicles, an also clear qualitative difference on the size of the endosomes was observed between *tbl* and control presynaptic endings (Fig. 8A to D). From seminal works of Heuser and Reese^23^ the endosomes located in the presynaptic endings have been related with the synaptic vesicles recycling. In present observations, quantitative analysis of the main components of the CLT recycling pathway^24–25^ demonstrated statistic significant differences on the number of CLT coated vesicles, endocytic pits, and endosomes. Thus, while control presynaptic terminals contained more coated vesicles, and endocytic pits than *tbl* ones (Fig. 8E); the number of endosomes was greater in *tbl* presynaptic boutons relative to control ones (Fig. 8E). Moreover, the qualitative appearance that *tbl* endosomes were bigger than control ones is clearly ratified by the measurement of their maximum diameters that were twice in *tbl* presynaptic endings relative to the control ones (Fig. 8F). This dysregulation of the recycling endocytosis pathway is reinforced by present immunocytochemical experiments. Early endosome antigen 1 (EEA1) distributed through the neuronal cytoplasm (Fig. 7A-B); and the quantitative analysis shows significant differences of the immunocytochemical expression of EEA1 between *tbl* and control cultured hippocampal neurons, being almost twice in *tbl* neurons regarding the control ones (Fig. 7D).

**Figure 9:**
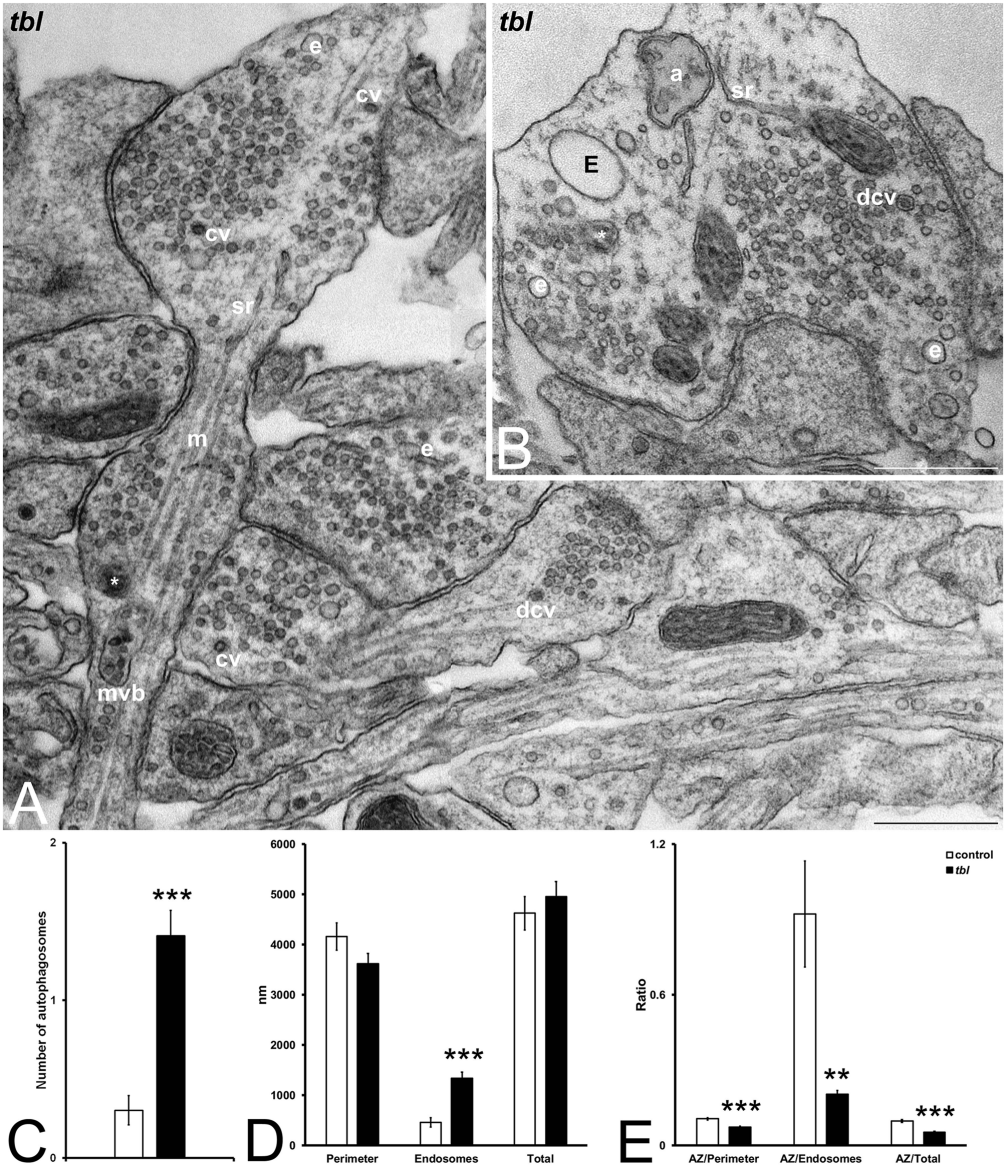
Electron microphotographs of *tbl* synapses (**A**–**B**) in which great endosomes (**E**) and autophagy vacuoles (a) are easily distinguished. Autophagosomes are most numerous in *tbl* than in control presynaptic terminals (**C**, ***p = 8.09153^E−05^). No differences stand out in the perimeter length of the presynaptic boutons (**D**, Perimeter; *p* = 0.06094) as well as in the perimeter of all membranes measured (**D**, Total; *p* = 0.22775) between control and *tbl* presynaptic boutons; however, the perimeter of the membrane of endosomes and autophagosomes (**D**, Endosomes) is significantly greater in *tbl* than in control presynaptic terminals (***p = 5.7819^E−07^). The ratios between the active zone and the perimeter length (**E**, AZ/Perimeter), the values of endodomes and autophagosomes membrane length (**E**, AZ/Endosomes), and the total length of measured membranes (**E**, AZ/Total) are consistently greater in control presynaptic terminals than in *tbl* ones (***p = 2,769^E−06^; **p = 0.0017; and, ***p = 1.8152^E−09^, respectively). cv, coated vesicles. dcv, dense core vesicles. e, endosomes. m, microtubules; mvb, multivesicular body. sr, smooth endoplasmic reticulum. Bars = 500 nm.

Another important finding of present experiments is related with the presence of autophagy vacuoles. Thus, from earliest reports, the ultrastructural features of these vacuoles as it is the presence of double membrane encircling damaged or ubiquitinated proteins^26^ help their easy identification. Autophagosomes and mitochondrial debris (Fig. 9B) are often observed in *tbl* presynaptic boutons; and, in addition to the previously described differences in the endosomes’ number and diameters between control cultured hippocampal neurons and *tbl* ones; the values of the counts of these vesicles showed an important increment of their number within *tbl* presynaptic terminals (n = 26) relative to control ones (n = 30) (Fig. 9C). From these observations it seems that the membrane dynamics of the presynaptic terminal might be altered in the *tbl* mutation. As the synaptic transmission takes place at the terminal active zone, which is smaller in *tbl* presynaptic terminal than in control ones (Fig. 1C-D), we have measured the perimeter of the presynaptic boutons, the perimeter of the endosomes and the perimeter of the autophagosomes and compared these in absolute values and as their ratios in relation to the active zone length (Fig. 9). Thus, while the values of presynaptic boutons perimeters and of the sum of the perimeter of the plasma membrane surrounding the bouton and that of the endo- and autophagosomes did not shown significant differences between control and *tbl* terminals (Fig. 9D), the perimeter of endosomes and autophagosomes was significantly greater in *tbl* terminals than in control ones (Fig. 9D). When the size of active zone is analyzed according their ratio respective to the values of the perimeter of the rest of presynaptic ending membranes were measured, there were significant differences between control and *tbl* presynaptic terminals in which these ratios were ever highest in control presynaptic boutons (Fig. 9E).

**Figure 10:**
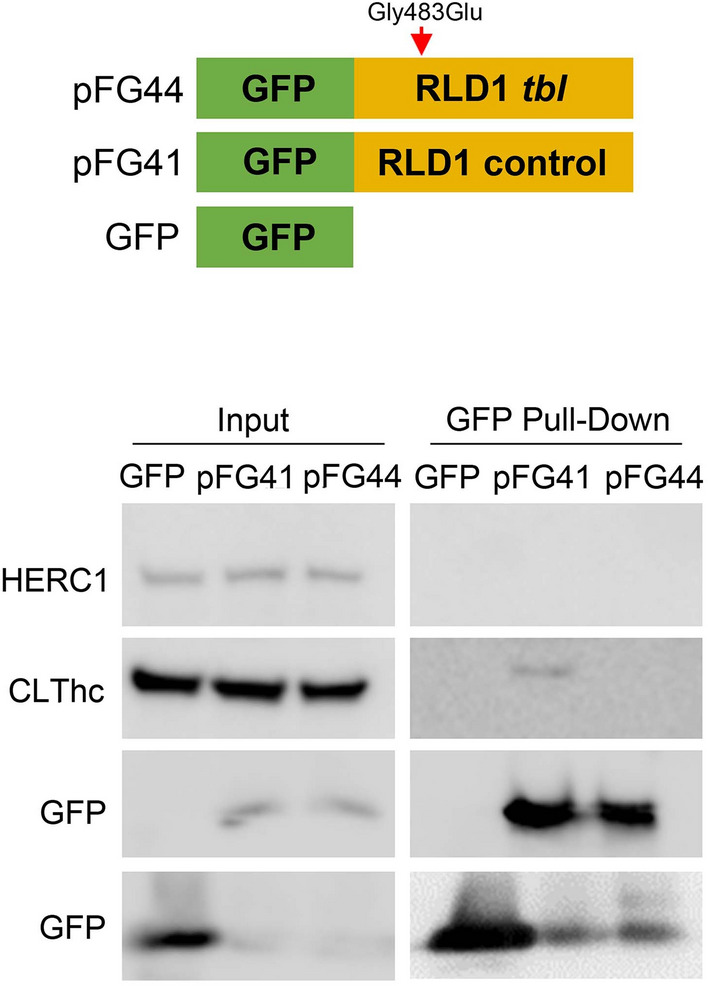
Impairment of the interaction between the RLD1 domain and the clathrin heavy chain (CLThc) by the *tambaleante (tbl)* mutation. GFP fusion proteins with the amino acid residues of HERC1 RLD1 domain are shown. The pFG44 construct (RLD1 *tbl*) has a point mutation, resulting in a Gly → Glu substitution at 483 amino acid residue of HERC1. Pull-down experiments were performed in HEK-293 T cells transfected with pFG41, pFG44 or GFP fusion constructs. After 72 h post-transfection, cell lysates were incubated with GFP-Trap Agarose resin for 2 h. Proteins retained in the resin were analyzed by immunoblotting using specific antibodies against the indicated proteins (see Supplementary information). Note the absence of interaction between HERC1 and pFG44 construct.

Although previous experiments described that HERC1-CLT interaction takes place through RLD2 domain^17^, we have tried to analyze the possible role that mutated RLD1 domain could play in this interaction. However, the fusion of proteins with amino acid residues of HERC1 RLD1 domain in cultured control and *tbl* hippocampal neurons experiments was not yet attained accurate levels in our hands. Therefore, as indirect analysis we have transfected HEK-293 T cells transfected with pFG41 (RLD1 control), pFG44 (RLD1 *tbl*) or GFP fusion constructs. 72-h post-transfection, the immunoblot of protein retained in the resin of the cell lysates demonstrated that in contrast to pFG41, pFG44 did not interact with CLT (Fig. 10, and Supplementary Information). Thus, this result shows for the first time that *tbl* RLD1 domain alters HERC1-CLT interaction, and open the possibility that this lack of interaction might be also present in *tbl* central synapses.

## Discussion

Biallelic *Herc1* mutation causes in humans a polymorphic syndrome with varied signs and symptoms^6–8^, together with intellectual disability [see table 1 in ref.^8^]. Recently a new *Herc1* mutation characterized by the synthesis of a truncated Herc1 protein lacking the C-terminal of HECT domain has been described, in addition with signs of the autism spectrum^27^. The animal model of *HERC 1* mutation, the *tambaleante* mouse, was first described as an example of adult cerebellar ataxia by autophagy Purkinje cell death^5,9–12^. Later studies showed more neurodevelopmental damages to *tbl* mice phenotype as: (1) delay in synaptic development and maturation of NMJ^16^; (2) anomalous myelination of peripheral nerves and delayed action potential transmission^15^; (3) increase of autophagy signs in projection neurons of central nervous system as the hippocampal CA3 pyramidal neurons, the neocortical pyramidal neurons, and the motor neurons of the spinal cord^13^; and, (4) impairment of associative learning linked to absence of LTP and anomalous spinogenesis of the neurons of the lateral amygdala^14^. Therefore, as part of the ubiquitin–proteasome system (UPS) whose alterations have been related with several neurodegenerative diseases of nervous system^28–34^, all these findings have been explained as the effect of the dysregulation of the autophagy^5,13–15^ and/or of the mammalian target of rapamycin complex 1 (mTORC1) altered regulation^5,15^. Present results in hippocampal cultured neurons show that *tbl* presynaptic terminals possess a noticeable decrease in the number of presynaptic terminals relative to the control ones, which in addition display: (1) a lower number of the ready releasable and the reserve pools of synaptic vesicles; (2) a shorter zone active; (3) less docked synaptic vesicles; (4) less SYT1; (5) less CLT coated vesicles and CLT immunoreactivity; (6) increased number of big endosomes correlated with and also high expression of EEA1; and, (7) an important presence of autophagosomes. These results are in accordance with previous findings in *tbl* mice in vivo. The decrease in the number of clear and round synaptic vesicles, in SYT1 expression as in the SV2B expression in *tbl* cultured neurons correlates with the decrease of SV2B-VGLUT1 co-expressing vesicles found in the lateral amygdala of adult *tbl* mice^14^. Furthermore, a diminution of the ready releasable pool of synaptic vesicles (RRP) together with a decrease in the probability of release was also found at the *tbl* NMJ^16^. Hence, all these data together strongly suggest that HERC1 might play a pivotal role in the presynaptic activity and the synaptic vesicle dynamics.

Postmitotic neurons are highly specialized cells whose presynaptic terminals lye several dozens or hundreds of microns far from the cell soma, and therefore part of the protein turnover resulting from the high synaptic activity takes place locally. Thus, more and more experimental evidences demonstrate that autophagy homeostasis, as well as their dysregulation eliciting neurodegenerative diseases, takes place at the presynaptic terminals^35–38^. In fact, studies in another model of ataxia involving Purkinje cell degeneration, the *Lurcher* mutant mouse, demonstrated that Purkinje cell axons react earlier and more strongly to autophagy than the Purkinje cell bodies^39^. Furthermore, *Lurcher* mice lacking the *Atg7* gene that encodes the autophagy-related protein 7 showed that axonal degeneration occurs prior to and independently of Purkinje cell death^40^. Indeed, it is now well established that axon terminals accumulate the greatest number of neuronal autophagosomes^41^ and that autophagy is key to preventing axonal degeneration after injury^42^.

The synaptic vesicle recycling pathways are far to be completely understood^43–45^. The endosomes play a pivotal role at least in two of these pathways: the CLT mediated endocytosis (CME), and the ultrafast endocytosis (UFE)^23,44^ provide: (1) the renewal of synaptic vesicles (CME and UFE); and/or (2) the elimination of damaged components of synaptic vesicles by lysosomal degradation (CME)^46^. Our results demonstrate an increase in the number of endosomes within the *tbl* presynaptic endings, which correlates to an also higher expression of EEA1 and with the presence of multivesicular bodies (Fig. 9). The clear deficit of CLT together to the reduction in synaptic vesicles number in *tbl* presynaptic endings could imply that less fused synaptic vesicles could integrate within the early endosomes, and could alter the complex process of endosomes maturation. In fact, alterations in the endosomes size and maturation have been reported in several neurological and neurodegenerative diseases [for a rev see^46^]. Therefore, our present findings open an interesting work line to analyze what of the proteins (CME interactors^47^, GTP-Rab interactions, i.e., see below) involved in the complex process of endosome maturation are altered by the HERC1 mutation. In fact, the role of CLT in the maintenance of synaptic vesicles number and in the endosomes homeostasis suggested by our observations, have been elegantly demonstrated by Kononenko et al.^48^, whose experiments with CLT KO mice, clearly demonstrated the depletion in synaptic vesicles together to endosome accumulation; two morphological data which are similar to that present the *tbl* presynaptic endings.

Several pathways might be dysregulated in the *tbl* mutation to control autophagy, proteostasis and the synaptic vesicle cycle^35–38^. These includes: (1) GTPases Rab family; (2) CLT; and (3) mTOR.

**Figure 11:**
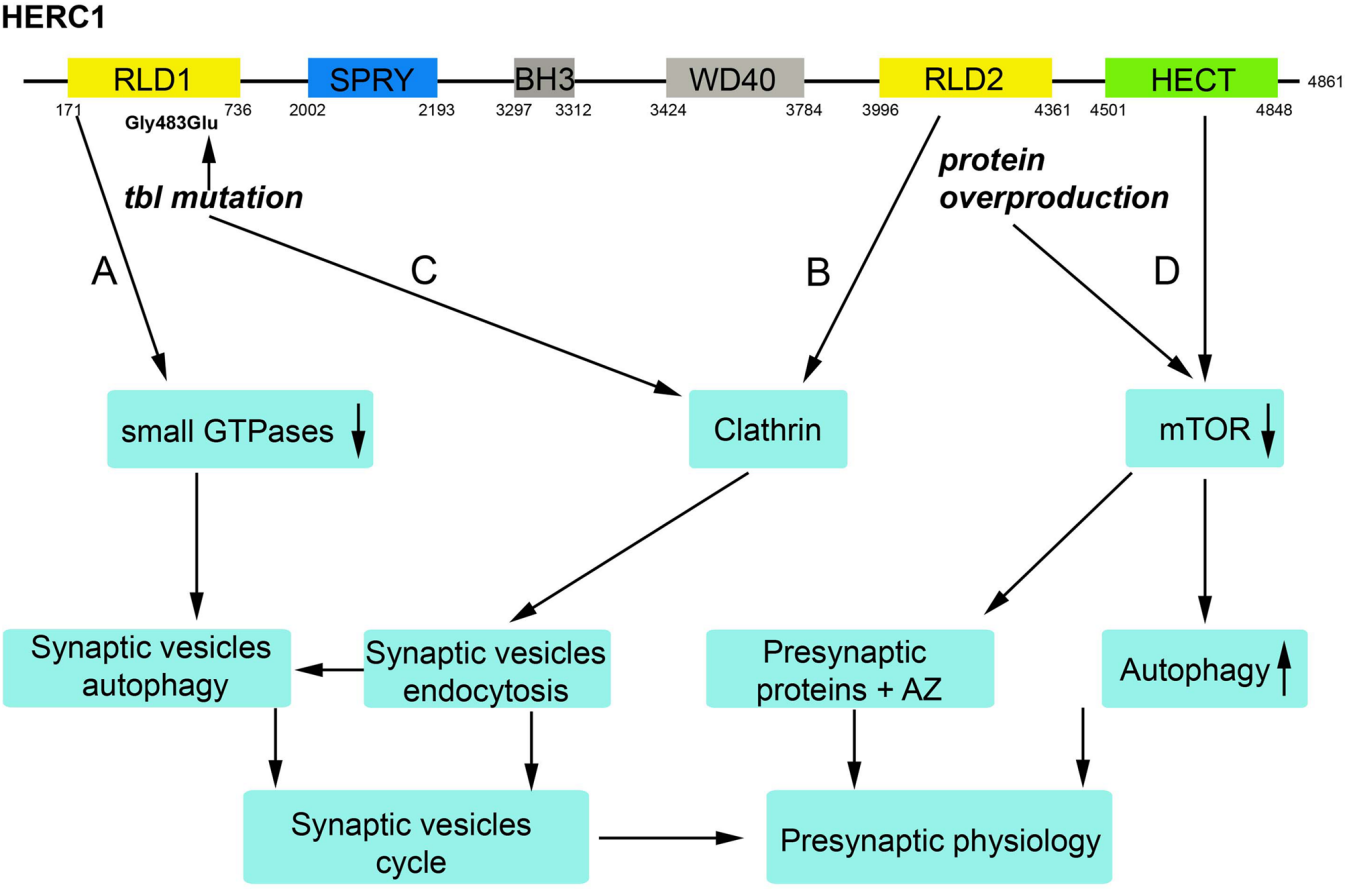
Schematic representation of the four pathways that the *tbl* mutation might dysregulate allowing to an increase of the autophagy of synaptic vesicles and the alteration on the number of healthy releasable synaptic vesicles. (**A**) Mutation site is located at RLD1 domain which dysregulating small GTPS might in turn dysregulate synaptic vesicles homeostasis. (**B**) RLD2 domain binds clathrin; therefore, alterations of RLD2 alone or by protein overproduction could alter endocytosis during synaptic vesicle recycling. (**C**) CLT-HERC1 interaction is impaired by mutated RLD1 and in turn could interfere the CME pathway. (**D**) mTORC1 activity decreased in *tbl* mutation and could thus explain the increase of autophagosomes; otherwise, mTORC1 participates in autophagy of presynaptic proteins whose dysregulation might contribute to the decrease in size of the active zone (AZ).

Amongst their many several functions, the GTPases Rab family has been involved in synaptic vesicles autophagy through the canonical endosomal-lysosomal system^36,49^. Moreover, the work of Binotti et al.^50^ demonstrated that Rab26 might be responsible for an independent pathway or *vesiculophagy* in which synaptic vesicles unable to recycle are redirected to the autophagosome in a non-canonical form. HERC1 RLD1 acts as a small GTPase regulator and inhibits GTPases Rab family^1,51^. Therefore, it could be conceivable that mutated RLD1 domain might alters the regulatory action of HERC1 on the GTPases Rab family affecting the mechanism of synaptic vesicles recycling (Fig. 11A).

CME is a key pathway for synaptic vesicles recycling at the presynaptic terminal^18,24,25^. CLT coated vesicles are uncoated by the action of the complex Endophilin A (Endo A)-Synaptojanin 1 (Synj 1)^25,51^. Synj 1 has been recently described as an autophagy regulator promoting the autophagosomes maturation^36,51,52^. Furthermore, a link between CLT coated vesicles and the autophagosome precursors has been described in basis to CLT-Atg 16 L1 (Autophagy related 16 like 1 protein) interaction^53,54^. HERC1 RLD2 interact with CLT and cytoplasmic vesicle trafficking^1,17^, and *tbl* mutation might alters CLT balance (Fig. 11B), as demonstrated by the decrease of CLT immunoreactivity and coated vesicles found in cultured hippocampal *tbl* neurons. These, correlate with the decrease in the number of synaptic vesicles and the increase in number and size of the endosomes in these neurons, indicative of a dysregulation of the synaptic vesicles recycling via CME. Whether the relationships between autophagy factors such Synj 1 or Atg 16 L1 should be further investigated to exclude that results found here are exclusively promoted by the CLT downregulation. Further, our GFP pull-down experiments in transfected HEK-293 T cells demonstrated that mutated HERC1 RLD1 domain was unable to interact with CLT (Fig. 11C), indicative that HERC1 mutation directly alters normal CLT dynamic^55^, irrespective of the role that such interaction will play through RLD2 domain, being suggestive that this disbalance could be also elicited in *tbl* central synapses.

The mTORC1 plays a key role in autophagy^56^, and its inhibition enhances autophagy, inducing a loss of dopaminergic synaptic vesicles^57^. In normal conditions, HERC1 could regulate mTORC1 activity through interaction with the tuberous sclerosis complex 2 (TSC2)^58^, helping thus to maintain the homeostatic autophagy; however, the activity of mTORC1 is reduced by HERC1 overproduction in the *tbl* mutation^5^, and this decrease in mTORC1 action could dysregulate autophagy and neuronal cell death found in *tbl* mice^5^. Therefore, the increase of autophagosomes and multivesicular bodies, and at least in part the decrease in the number of synaptic vesicles could be explained by a misfunction of the HERC1 protein in *tbl* mutation (Fig. 11D).

In conclusion, although the specific targets and pathways damaged by the HERC1 mutation are still unsolved, our results ratify and extend previous in vivo findings and leads us to hypothesize that HERC1 mutation alters central synapses dynamic by impairing the normal CLT balance, altering thus the homeostasis of synaptic vesicles recycling. Therefore, we postulate HERC1 as an important regulator in neurodevelopment, and particularly in the alterations of synaptic transmission homeostasis, one of the bases of the intellectual disability.

## Materials and methods

### Animals

*Tambaleante* mice were obtained by breeding pairs of the *tbl* carrier mice, genotyping the offspring by PCR^5^ at birth (P0). The animals were handled in accordance with current Spanish and European legislation governing the use of experimental animals (RD 53/2013—BOE 08/02/2013 and 2010/63/EU), and all experimental procedures were approved by the University of Seville and Pablo de Olavide University ethics committees, and the Junta de Andalucía (Animal Health Service auth. # 13/06/2017/080).

### Cell culture

Hippocampal neurons from control and *tbl* P0-P1 mice were cultured as described previously^20^. Every set of experiments were performed in littermates. CA1 and CA3 hippocampal regions from both sides were isolated and removed from the brain of P0–P1 newborn mice. The two hippocampi were immersed in 1 ml of enzymatic solution and kept under continuous agitation (80 rpm) for 40 min and centrifuged at 200×g for 5 min. The enzymatic solution was previously bubbled with 95% O_2_ and 5% CO_2_ for 20 min, and contained 1 ml of DMEM (PAA Laboratories), 0.2 mg of cysteine, 1 mM CaCl_2_, 0.5 mM EDTA and 5 units/ml papain (Worthington). After centrifugation, the supernatant was removed, and 1 ml of inactivating solution [800 μl of DMEM (Gibco), 200 μl FBS (BioWest), 200 units of penicillin (Gibco), 200 μg of streptomycin (Gibco), 2.5 mg albumin and 2.5 mg trypsin inhibitor (Sigma)] was added to the tissue to prevent further digestion. Thereafter, the tube was centrifuged again at 200 × g for 5 min and mechanically dispersed afterward with a 1 ml pipette tip five times. The supernatant was discarded and replaced with neuronal media, containing: 1 ml Neurobasal (Gibco), 20 μl B27 (Gibco) (50 ×), 10 μl Glutamax (Gibco) (100 ×), 20 units penicillin and 20 μg streptomycin. Neurons were plated at a density of 150,000 cells/well (12-well plates) over a single feeding layer of astrocytes grown on 15 mm ϕ coverslips^20^. Neurons were cultured between 14–17 DIV in an incubator at 37 °C with an atmosphere of 95% O_2_ and 5% CO_2_.

### Transmission electron microscopy (TEM)

Coverslips containing hippocampal neuronal cultures were fixed with 2% paraformaldehyde, 2.5% glutaraldehyde and 0.02% CaCl_2_ in 0.1 M cacodylate buffer (pH 7.3) (CB) for 1 h at 4 °C. Thereafter the cultures were treated with 2% osmium tetroxide in CB, 1% uranyl acetate in 70% ethanol, dehydrated with an increasing gradient of ethanol and embedded in Durcupan (Fluka^®^). Ultrathin Sects. (50–70 nm thick) were cut with a Leica UC6 ultramicrotome, collected in copper grids (150 and 300 mesh) and one-slot formvar coated grids, and observed by TEM without counterstaining on a Zeiss Libra microscope at 80 kV (CITIUS, University of Seville).

For synaptic vesicles count w^®^e have followed the methodology proposed by Schikorski and Stevens^59^. Presynaptic endings (control n = 30; and *tbl* n = 26) were divided in four compartment (75 nm width each) parallels to the active zone. All counting was done with the Fiji ImageJ software (W. Rasband, National Institutes of Health, https://imagej-nih.gov/ij/). Parameters considered here were: (1) the total number of vesicles; (2) the active zone length; (3) the synaptic vesicles diameter; (4) the intervesicular distance; (5) the number of docked vesicles; (6) the number of tethered vesicles; (7) the number of synaptic vesicles for each 75 nm width compartments; (8) the number of CLT coated vesicles; (9) the number of endocytic pits; (10) the number of endosomes; and (11) the maximum diameter of the endosomes.

### FM1-43 loading

On the day of the experiment the coverslips were brought from the incubator, and the media solution exchanged with the recording solution (in mM: 140 NaCl, 2.4 KCl, 10 HEPES, 4 CaCl_2_, 4 MgCl_2_, 10 glucose, pH 7.3, osmolality 305 mmol/kg). Neurons were used for up to 45 min without superfusion. All chemicals used were from Sigma-Aldrich, except the culture media products (GIBCO), and papain (Worthington).

Synaptic terminals were loaded by substitution of basal solution with 70 mM [K^+^] Locke solution (generated by equimolar replacement of NaCl by KCl) containing 4 μM FM1-43 (Molecular Probes) for 2 min. For imaging experiments, we use an inverted microscope IX-71 (Olympus) located on an isolation table. The recording chamber was set up on a separate XY stage attached to an optical bench column. A 3-axis mechanical manipulator was also attached for micropipette positioning. The microscope camera port allows 100% light reflection to which a QE-Sensicam camera (12 bit, PCO) was attached. The light source was a monochromator polychrome IV (100 W Xenon lamp) (Till Photonics). Image acquisition and illumination were controlled with TILL Vision software. Images were collected through a plan-NeoFluor 40 × objective (0.75 NA). Excitation of the FM1-43 was done at 488 nm and the emission light was collected through a band-pass emission filter (510 ± 10 nm). The pixel size was 0.32 μm. Neurons were stimulated by placing a micropipette near the soma (≈ 5–10 μm). Micropipettes were pulled in a P97 (Sutter Instruments) puller, having a resistance between 1–2 MΩ, when filled with the recording solution. Neuronal stimulation was done with an electrical stimulator (model 2,100, AM-Systems) by passing biphasic 1 ms pulses of 45–50 μA. A TTL signal generated with the TILL Vision software triggered stimulation and data acquisition. We collect 250 images per experiment. Exposure time was 800 ms, the image sampling rate was 1 frame/s. We used Igor Pro 6.22 (Wavemetrics Inc) custom made macros for image analysis. Synaptic responses were identified as regions the destaining of fluorescence. The ROIs were a square of 5 × 5 pixels (1.6 μm by 1.6 μm for a 40 × objective). For analysis, we use a Kolmogorov–Smirnov to check the normality of the samples and apply a *t *test to compare mean values.

### Immunocytochemistry and quantification

Hippocampal cultures were fixed with 4% paraformaldehyde in 0.12 M phosphate buffer (pH 7.2–7.4). After fixation, the cultures were washed in PBS, and immunolabelled using the procedure reported previously^13–14^ The primary antibodies used were: a mouse monoclonal antibody against clathrin (CLT) light chain (1:500, Synaptic Systems, #113,011); a mouse monoclonal antibody against early endosome antigen 1 (EEA1) (1:50, ThermoFisher,MA-14794); a rabbit polyclonal antibody against HERC1 (1:150, ThermoFisher,PA5-62,033); a rabbit polyclonal antibody against microtubule associated protein 2 (MAP2) (1:500, Synaptic Systems, #188,003); a rabbit polyclonal antiserum against the presynaptic vesicle protein SV2B (1:200, Synaptic Systems, #119,102); and a mouse monoclonal antibody against synaptotagmin 1 (SYT1) (1:100, Synaptic Systems, # 105,011). The secondary antibodies used were: a donkey anti-rabbit IgG (H + L) highly cross-adsorbed antibody, Alexa Fluor 488 (1:500, ThermoFisher, #A-32790); a donkey anti-rabbit IgG (H + L) highly cross-adsorbed antibody, Alexa Fluor 555 (1:500, ThermoFisher, #A-31572); a donkey anti-mouse IgG (H + L) highly cross-adsorbed antibody, Alexa Fluor 555 (1:500, ThermoFisher, #A-31572); and a goat anti-mouse IgG (H + L) cross-adsorbed antibody, Alexa Fluor 488 (1:500, ThermoFisher, #A-11001).

The images were acquired on an upright Olympus FluoView 1,000 confocal laser scanning microscope. The figures were prepared using the Photoshop 7.0 (Adobe^®^) software without any additional correction.

Immunoreactivity was quantified as indicated previously^14^. Briefly, an alternating sequence of laser pulses was used to activate the different fluorescent probes during image acquisition. Images were acquired with a 60 × oil-immersion objective (N.A. 1.42). Images from the hippocampal cultures of control and *tbl* mice were obtained in the same session under similar conditions (laser intensities and photomultiplier voltages). Quantification of the fluorescent labelling intensity was performed offline with ImageJ and the size of the areas measured was determined automatically by defining outline masks based on the brightness thresholds from maximal projected confocal images. The control and *tbl* images of CLT, EEA1, HERC1, MAP2, SV2B and SYT were captured as follows and expressed in arbitrary units: (1) CLT: Rhodamine laser intensity 5% with photomultiplier settings HV 410, Gain 2, Offset 25; (2) EEA1: FITC laser intensity 4% with photomultiplier setting HV 530, Gain 3., Offset 15; (3) HERC1: Rhodamine laser intensity 1% with photomultiplier setting HV 600, Gain 3, Offset 13; (4) MAP2: FITC laser intensity 10% with photomultiplier setting HV 413, Gain 3, Offset 10; (5) SV2B: FITC laser intensity 5%, with photomultiplier settings HV 401, Gain 3, Offset 15; and (6) SYT1: FITC laser intensity 4% with photomultiplier setting HV 530, Gain 3., Offset 15.

### Plasmids and pull-down experiments

pFG41 construct (GFP-RLD1 fusion containing 365–794 amino acid residues of HERC1) was previously described^60^. pFG44 construct was similar to pFG41 but containing the punctual mutation Gly483Glu found in *tbl* animals. This construct was generated from pFG41 using QuickChange XL Site-directed mutagenesis kit (Stratagene) following instructions of manufacturer. For the GFP pull-down, supernatants were incubated with 2μL of GFP-Trap© Agarose resin (Chromotek, Germany) around 2 h at 4 °C. After the incubation, the samples were centrifuged (2,500 g) and pull-downs were washed with NP40 buffer (three times). After each wash the samples were centrifuged again. Pellets were analyzed by electrophoresis and immunoblotting by using the following antibodies: anti-HERC1 (410)^55^, anti-GFP (Abcam), and anti-clathrin (CLT) heavy chain (hc) (BDBiosciences).

### Statistical analysis

The statistical analyses of the data from the TEM, FM1-43 destaining, and immunolabelling experiments were analyed blind by EMP-V and MAM-F. A two tailed Student’s *t* test was used to compare the data from *tbl* and control hippocampal cultures. Any *p*-values less than 0.05 were considered significant, indicated as follows: * *p* < 0.05, ** *p* < 0.01, and *** *p* < 0.001.

## Supplementary information

Supplementary information
