# Supplementary material for: The HERC1 ubiquitin ligase regulates presynaptic membrane dynamics of central synapses
Source: Sci Rep. 2020 Jul 21;10:12057. doi: 10.1038/s41598-020-68970-8 (PMC7374096; doi:10.1038/s41598-020-68970-8)
Supplement: Supplementary file 1 — Supplementary information [file 41598_2020_68970_MOESM1_ESM.pdf]

The HERC1 ubiquitin ligase regulates presynaptic membrane dynamics of central synapses

Mª Angeles Montes-Fernández[1¶], Eva Mª Pérez-Villegas[2¶], Francesc R. Garcia-Gonzalo[3], Leonardo Pedrazza[3], Jose Luis Rosa[3], Guillermo Alvarez de Toledo[1*], José A. Armengol[2*]

[1]Department of Medical Physiology and Biophysics, School of Medicine, University of Seville, Seville, Spain

[2]Department of Physiology, Anatomy and Cell Biology, University Pablo de Olavide, Seville, Spain.

[3]Department of Physiological Sciences, IDIBELL, University of Barcelona, Barcelona Spain

* Corresponding authors

E-mail: jaarmbut@upo.es (JAA)

E-mail: gat@us.es (GAT)

¶ These authors contributed equally to this work.

RAW DATA

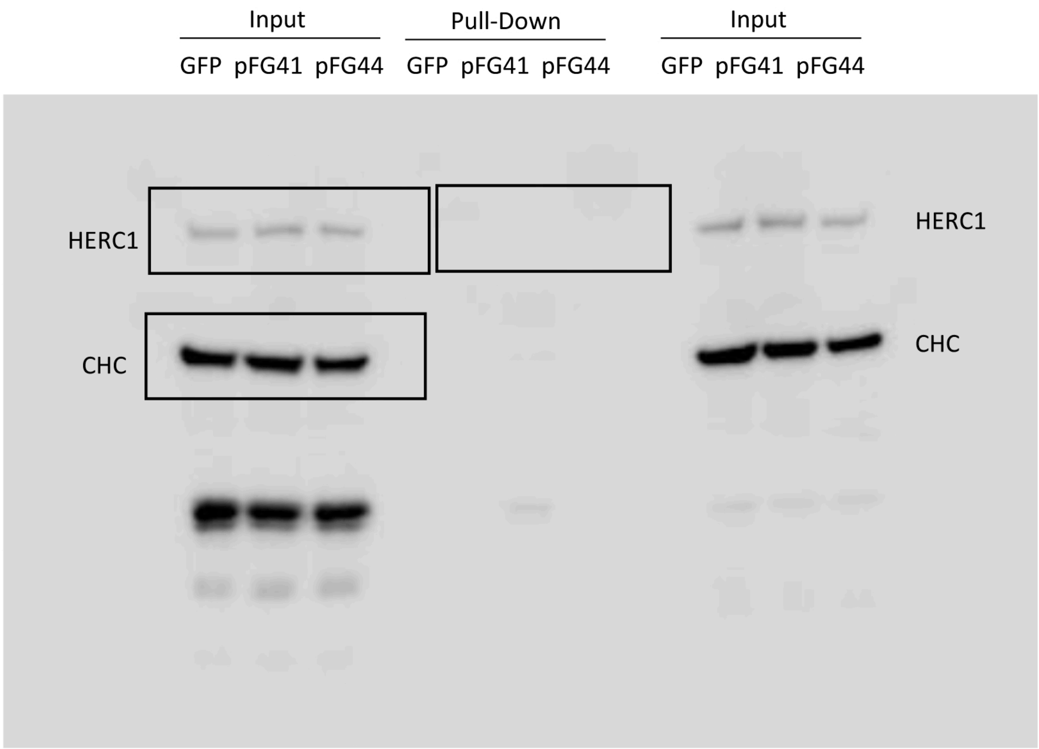
Input
Pull-Down
Input
GFP pFG41 pFG44
GFP pFG41 pFG44
GFP pFG41 pFG44
HERC1
CHC
HERC1
CHC

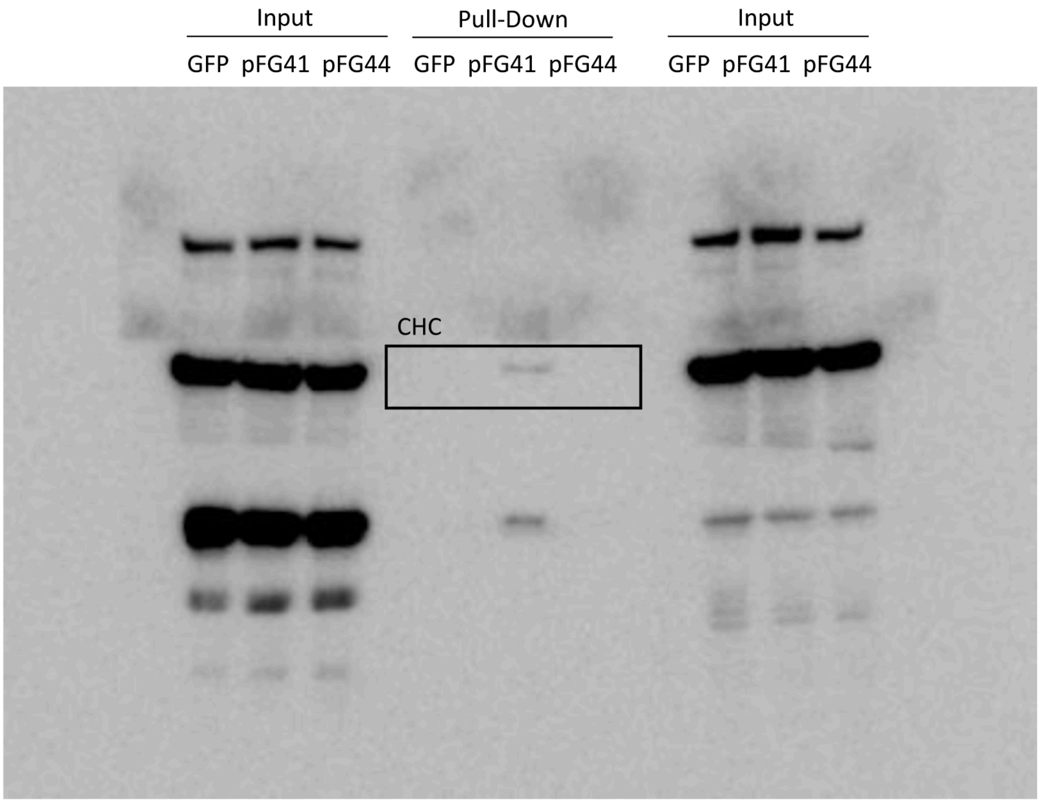
Input
Pull-Down
Input
GFP pFG41 pFG44
GFP pFG41 pFG44
GFP pFG41 pFG44
CHC

RAW DATA

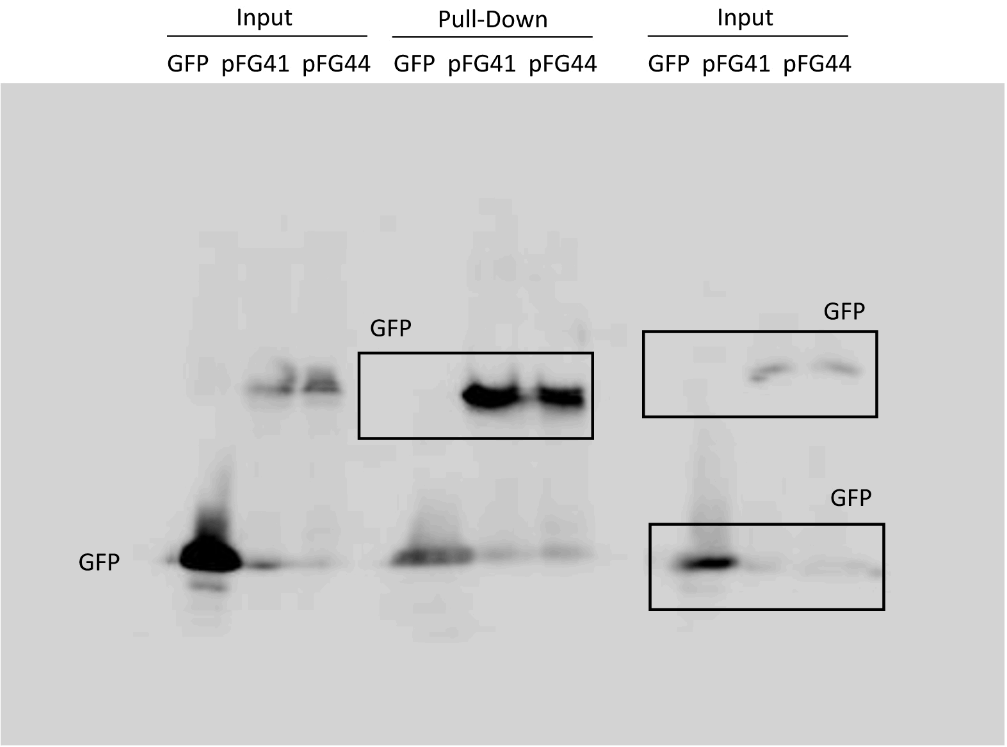
Input
Pull-Down
Input
GFP pFG41 pFG44 GFP pFG41 pFG44 GFP pFG41 pFG44
GFP
GFP
GFP
GFP

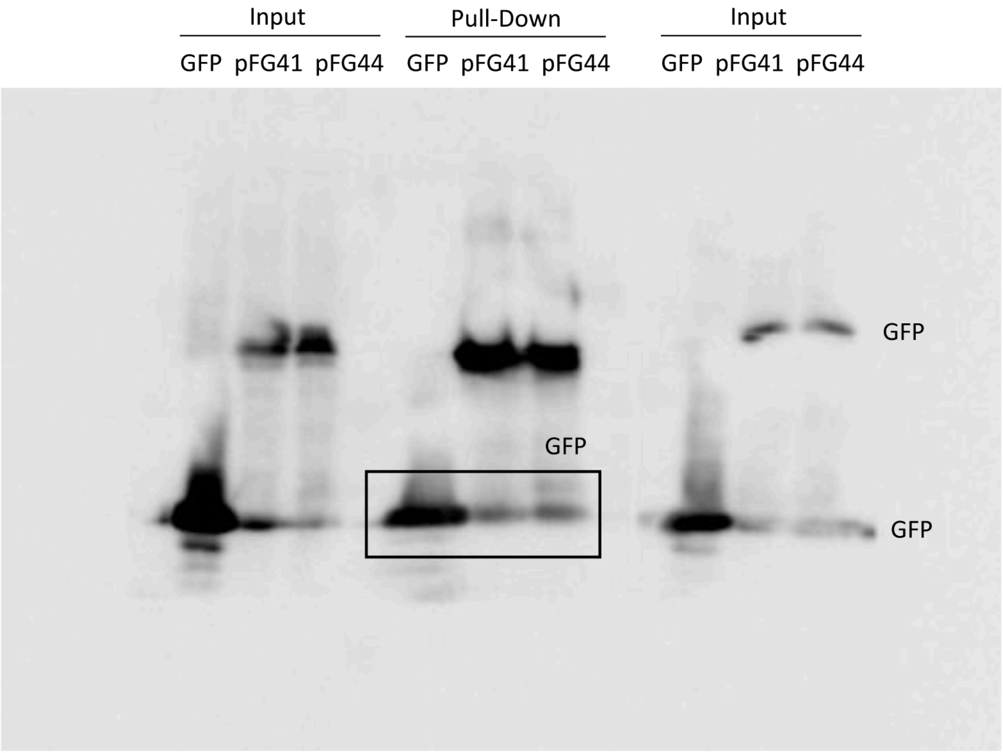
Input
Pull-Down
Input
GFP pFG41 pFG44 GFP pFG41 pFG44 GFP pFG41 pFG44
GFP
GFP
GFP
